# 809. Exploring the Relationship Between Burn Injury and COVID-19 Infection

**DOI:** 10.1093/jbcr/irag033.187

**Published:** 2026-04-06

**Authors:** Victoria Teoh, Kaitlin Nunn, Zeyanna Dhalla, Amina El Ayadi, Steven E Wolf, Juquan Song

**Affiliations:** University of Texas Medical Branch, Galveston, TX; University of Texas Medical Branch, Galveston, TX; University of Texas Medical Branch, Galveston, TX; Division of Burn and Trauma Surgery, Department of Surgery, University of Texas Medical Branch, Galveston, Galveston, TX; Division of Burn and Trauma Surgery, Department of Surgery, University of Texas Medical Branch, Galveston, Galveston, TX; University of Texas Medical Branch, Galveston, TX

## Abstract

**Introduction:**

Burn injuries and COVID-19 represent two distinct disease etiologies and pathologies. Although both conditions share traits of unresolved inflammation, altered biogenetics, and chronic functional impairment, the correlation between immune profile and inflammatory response is not clear. This study aims to investigate the potential similarities between COVID and burn injury based on inflammatory markers such as monocytes, lymphocytes, procalcitonin (PCT), and C-reactive Protein (CRP).

**Methods:**

A retrospective cohort study was conducted using TriNetX, a multi-institutional database of electronic health records. Data was collected from 2024-2025 to evaluate non-immunosuppressed adult patients (18-65) with either a symptomatic COVID infection or a 10-50% TBSA burn injury. The primary outcomes evaluated were CRP, lymphocytes, monocytes, and PCT over time intervals from 0-120 days. Propensity score matching and statistical analysis were done using standardized mean differences (SMD). An SMD < 0.2 is notably similar, whereas >0.2 represents a slight imbalance between groups.

**Results:**

Across all time intervals, all SMD values for lymphocytes, monocytes, and PCT remained <0.2 between burns and COVID. The monocyte count was the most comparable value between the two groups, with minimal standardized mean differences (≤ 0.096), with no extreme divergence between conditions. While CRP was initially similar (0-3 days, SMD ≈ 0.04, and 8-30 days SMD ≈ 0.055), its levels also showed the greatest and most sustained differences, peaking at 31-60 days (SMD ≈ 0.28).

**Conclusions:**

This study identified potentially shared immune signatures between burn injury and symptomatic COVID. Lymphocyte and monocyte SMD values remained <0.20 at all time intervals, reflecting similar adaptive and innate cellular responses, respectively, in burns and COVID. The innate markers, CRP and PCT showed SMD values >0.2, demonstrating slight differences likely due to the persistent sterile injury in burns and a resolving viral infection in COVID. Despite the differing mechanisms, there are substantial similarities between the immune responses. Further research into the damage and pathogen-associated molecular patterns could clarify the potential overlap between the COVID and burn immune responses.

**Applicability of Research to Practice:**

By recognizing the mechanistic overlap between these conditions, clinicians can accelerate therapeutic reach and design interventions to address the shared burden of chronic inflammation. Clinicians can potentially utilize established COVID interventions toward burn care.

**Funding for the study:**

N/A.

